# Epidemiology of bacterial biofilms on polyps and normal tissues in a screening colonoscopy cohort

**DOI:** 10.1080/19490976.2025.2452233

**Published:** 2025-01-18

**Authors:** Julia L. Drewes, Samara B. Rifkin, Madison McMann, Sara Glass, Emma Spence, Caroline R. Wensel, Abby L. Geis, Courtney Stevens, Joell J. Gills, Hao Wang, Linda M. Hylind, Gerard Mullin, David Kafonek, David Cromwell, Louis La Luna, Francis M. Giardiello, Cynthia L. Sears, the Biofilm Study Consortium

**Affiliations:** a Division of Infectious Diseases, Department of Medicine, Johns Hopkins University School of Medicine, Baltimore, MD, USA; b Bloomberg-Kimmel Institute of Immunotherapy, Johns Hopkins University School of Medicine, Baltimore, MD, USA; c Department of Oncology, Sidney Kimmel Comprehensive Care Center, Johns Hopkins University School of Medicine, Baltimore, MD, USA; d Department of Gastroenterology, University of Michigan, Ann Arbor, MI, USA; e Division of Gastroenterology, Department of Internal Medicine, John D. Dingell VA Medical Center and Wayne State University School of Medicine, Detroit, MI, USA; f Division of Gastroenterology and Hepatology, Department of Medicine, Johns Hopkins University School of Medicine, Baltimore, MD, USA; g Green Spring Endoscopy, Lutherville, MD, USA; h Maryland Endoscopy Center, Towson, MD, USA; i Digestive Disease Associates, Reading, Wyomissing, PA, USA; j Department of Pathology, Johns Hopkins University School of Medicine, Baltimore, MD, USA; k Johns Hopkins University School of Medicine and Green Spring Endoscopy, Lutherville, MD; l Johns Hopkins University School of Medicine, Baltimore, MD; m Reading Hospital, Tower Health, Reading, PA; n Digestive Disease Associates, Reading, Wyomissing, PA

**Keywords:** Biofilms, colonoscopy, bowel preparation, colorectal cancer, polyps

## Abstract

**Background:**

Invasive bacterial biofilms are implicated in colorectal cancer. However, their prevalence on histologically normal tissues and polyps is not well established, and risk factors of biofilms have not been previously investigated. Here we evaluated potential procedural and demographic risk factors associated with biofilm status using a cross-sectional observational cohort.

**Methods:**

Histologically normal colonic biopsies from 2,051 individuals undergoing screening colonoscopy were evaluated for biofilm status using fluorescence in situ hybridization with oligonucleotide probes targeting bacterial 16S rRNA. Polyp tissues from 21 individuals were also examined. Procedural, demographic, and lifestyle predictors of bacterial scores were evaluated using multivariable proportional odds regression models.

**Results:**

Procedural variables that negatively impacted bacterial scores and biofilm detection included smaller biopsy forcep size, physician, bowel preparation type, and shorter times from bowel preparation completion to colonoscopy. Lifestyle variables including greater alcohol and cigarette usage were associated with higher bacterial scores, while vigorous physical activity and diabetes mellitus were associated with lower bacterial scores. Bacterial scores on normal tissues were significantly higher in individuals with colorectal cancer but not polyps compared to dysplasia-free individuals. Direct examination of polyp tissues demonstrated similar bacterial burden and taxonomic composition compared to paired normal tissues, but polyps displayed enhanced bacterial invasion into crypts. Additionally, bacterial scores significantly correlated with increasing polyp size, suggesting co-evolution of polyps with bacterial invasion and biofilm status.

**Conclusions:**

Colonic biofilms are highly dynamic ecosystems that associate with several other known risk factors for colorectal cancer. However, biofilm detection is impacted by multiple procedural factors.

## GRAPHICAL ABSTRACT

**Figure:**
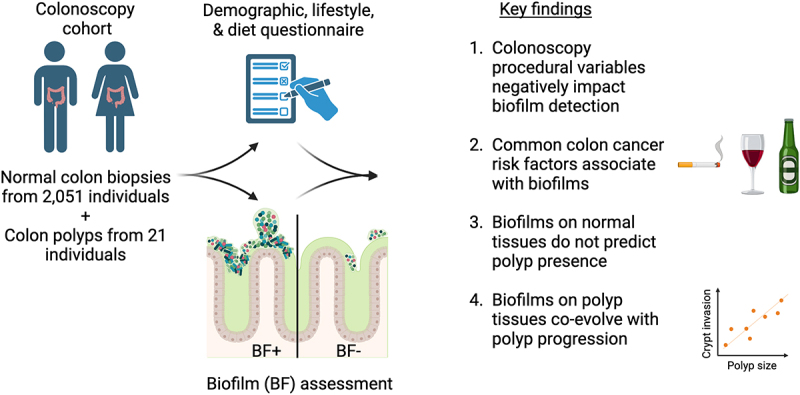

## Introduction

Cross-sectional cohort studies have demonstrated an association between the presence of mucus-invasive, colonic bacterial biofilms and gastrointestinal (GI) disorders including inflammatory bowel disease (IBD) and colorectal cancer (CRC). ^1–6^ Invasive colonic bacterial biofilms occur when the normally sterile inner mucus layer is penetrated by luminal bacteria, enabling direct interactions between the colonic epithelium and bacteria, bacterial products, and virulence factors that are hypothesized to contribute to the pathogenesis of GI disease.^7^ For example, colonic mucosal biofilms are associated with altered inflammatory states even in healthy individuals,^1,8^ and inoculation of human biofilm-positive colonic tissue slurries into germ-free mice led to rapid biofilm formation and enhanced tumor formation in mice heterozygous for the *Apc* gene (multiple intestinal neoplasia or *Apc*^*Min/+*^ mice).^9,10^ However, our understanding of biofilm epidemiology at the pre-cancerous stage remains limited. In this study, we examined the association of biofilms with polyp status as well as the impact of procedural and demographic/lifestyle factors on bacterial scores in a screening colonoscopy cohort.

## Materials & methods

### Colonoscopy cohort

**Figure 1.**
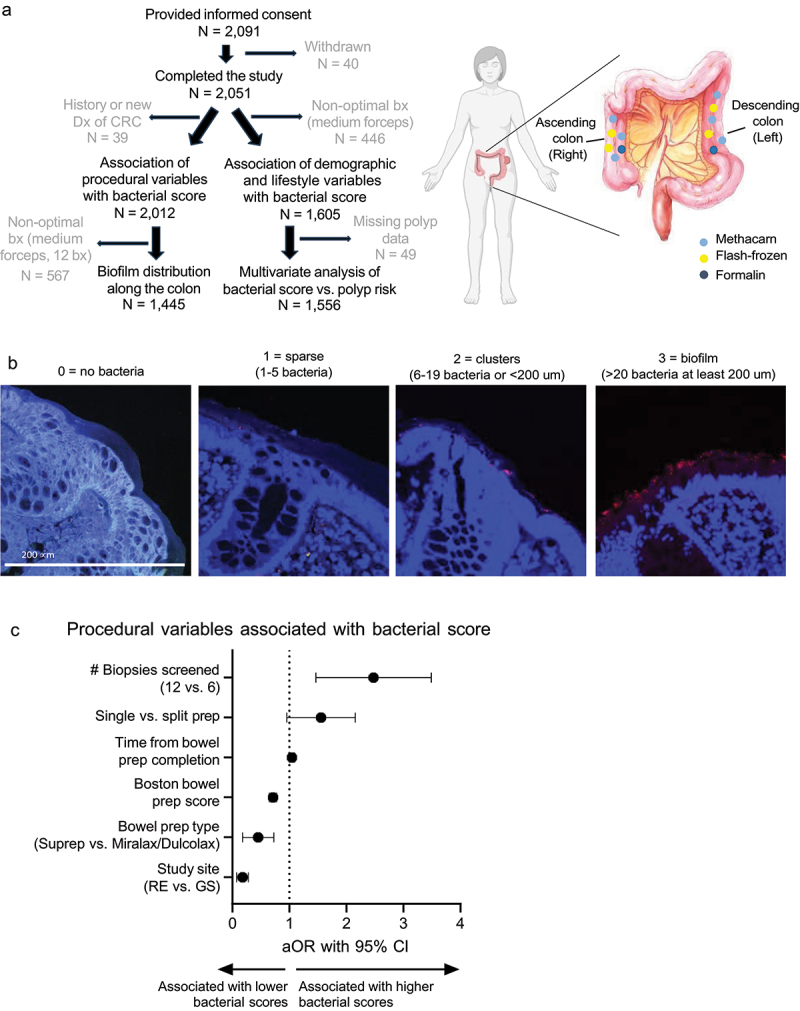
Bacterial biofilm scoring. (a) Flow chart of colonoscopy study design and analysis. (b) Representative biofilm scoring on 0–3 scale. Biopsies were stained with the EUB338 universal probe (red) and DAPI (blue) and imaged by confocal microscopy at 40X. (c) Forest plot of procedural factors influencing bacterial score on methacarn-fixed colonic biopsies. Adjusted odds ratios (aOR) with 95% CI from a proportional odds logistic regression model are shown.

Individuals undergoing routine preventive screening, surveillance, and/or diagnostic colonoscopy were recruited from three endoscopy study sites between August 2016 and March 2020: Green Spring Endoscopy, Lutherville, MD; White Marsh Endoscopy Center, Baltimore, MD; Reading Endoscopy Center, Wyomissing, PA. The study was reviewed and approved by the Johns Hopkins Medical Institute (JHMI) IRB for human research (IRB00094020). The Digestive Disease Associates/U.S. Digestive Health and Reading Hospital IRB, a subsidiary of the Johns Hopkins IRB, also approved the study. An internal data safety and monitoring committee also oversaw the study. Inclusion criteria were adult outpatients undergoing elective colonoscopy. Exclusion criteria included IBD, warfarin or antiplatelet drugs, and pregnancy. A total of 2,091 individuals were enrolled (~40% of all eligible per prior estimates,^11^ and 2,051 completed the study (Figure 1a).

Participants underwent colonoscopy following bowel preparations according to their physician recommendations (Table S1). Biopsies from the ascending colon (6) and then descending colon (6) were acquired with medium or large capacity biopsy forceps (Olympus or Boston Scientific). Three biopsies per side were fixed in modified methacarn (6:3:1 ratio of methanol:acetic acid:chloroform) at 4 ${\;}^{\circ }C$ for at least 24 h. The remaining biopsies were formalin- fixed or flash-frozen. For a subset of individuals, all 12 biopsies (6 ascending, 6 descending colon) were fixed in modified methacarn to determine the impact of additional sampling on biofilm rates (*N* = 107 Reading, 10 Greenspring, 10 White Marsh enrollees). Following fixation, samples from the Reading, PA site were shipped overnight via FedEx to Johns Hopkins. All fixed samples subsequently underwent three gentle washes in either 100% methanol (for methcarn-fixed tissues) or 70% ethanol (for formalin-fixed tissues) followed by paraffin embedding at Johns Hopkins.

Information on demographics, medical and surgical history, medication use, family history of CRC, patterns of tobacco use, alcohol use and physical activity, and history of prior colonoscopy were obtained via questionnaire administered by study personnel. Participants self- reported diabetes mellitus and hyperlipidemia status. Diet was investigated with a short dietary questionnaire, adapted from the questionnaire used by Hazen and colleagues.^12^

### Biofilm screening

Methacarn- and FFPE sections were stained by fluorescence *in situ* hybridization (FISH) as previously described with the all-bacterial probe EUB338 (Table S2) and DAPI.^2,13^ Samples were scored for bacterial biofilm formation by two independent screeners in a blinded, randomized manner on a Nikon E800 epifluorescent microscope at 40X objective with a 10X eyepiece. Mucus-invasive bacteria were scored on a scale from 0 to 3 in a 200 µm expanse of colonic epithelial cells (CEC) with the most adherent bacteria (Figure 1b), counting only EUB338-positive bacteria within 1 µm of CECs: 0 = no bacterial invasion, 1 = sparse invasion with 1–5 bacteria, 2 = clusters/moderate invasion with 6–19 bacteria, and 3 = continuous biofilm spanning > 20 bacteria.^1,3^ Bacterial scores <2.5 were confirmed by consensus of the two screeners on a Zeiss 780 laser scanning confocal microscope. Individuals with at least one biopsy scored as 3 were designated biofilm positive. Additionally, scores from all biopsies per individual were averaged to generate an average bacterial score. In total 12,948 methacarn-fixed normal biopsies, 18 formalin-fixed normal biopsies, and 49 formalin-fixed polyps were screened by FISH with the EUB338 probe and DAPI (13,015 samples total).

### Multi-probe FISH

A subset of samples scored as biofilm positive were subsequently stained by multi-probe FISH with taxa-specific probes (Sigma-Aldrich, USA) and DAPI as previously described (Table S2). ^14–17^ Samples were pre-screened using whole slide imaging on an Akoya Vectra Polaris imager at 20X in the Tumor Microenvironment Core (TME) at the Johns Hopkins University School of Medicine (JHU SOM) followed by high-resolution 40X images on a Zeiss 780 confocal microscope with linear unmixing using lambda scanning technology in the JHU SOM Microscope Facility Core.

### Mucus screening

Mucus visible via autofluorescence on FISH-stained slides was scored on a scale from 0–2: 0 = no areas with screenable, attached mucus; 1 = multiple regions of screenable mucus spanning up to 50% of the colonic epithelium; 2 = samples with mucus preserved on > 50% of the colonic epithelium. Mucus quality for a subset of methacarn-fixed biopsies was confirmed by Alcian Blue and Nuclear Fast Red staining per the manufacturer’s instructions (Millipore Sigma). Slides were imaged on a Hamamatsu Nanozoomer slide scanner in the JHU SOM TME Core. Average mucus thickness per biopsy was measured in a blinded, randomized fashion in FIJI by measuring the thickness at 50 µm intervals across three 200 µm regions of mucus.

### Polyp analyses

Location, size, and diagnosis of colorectal polyps were abstracted from medical records and colonoscopy reports to stratify study participants. Hyperplastic polyp (HP) = only HP. Adenomatous polyp (AP) = one or more tubular, tubulovillous, or villous AP ± dysplasia and ± synchronous HP. Sessile serrated polyp (SSP) = one or more SSP, ± synchronous HP. Synchronous cases had both APs and SSPs ± hPs. Due to their rarity, traditional serrated adenomas were excluded from this analysis (*N* = 5). Advanced adenomas = polyps ≥1 cm, containing villous or dysplastic components, or > 3 polyps. Individuals without polyps had a complete colonoscopy with visualization of the cecum without any evidence of polyps. Slides from FFPE polyps from a subset of study participants were obtained from the Johns Hopkins Hospital Pathology tissue bank in accordance with HIPAA and IRB regulations.

### Statistics

Pair-wise statistics were performed in GraphPad Prism (v8). The bacterial scores did not follow a normal distribution by Shapiro-Wilk’s test for normality. Therefore, for the multivariable analyses, we created ordinal values by binning continuous bacterial scores (0, > 0 to < 1, ≥1 and < 2, and ≥2) into a 4-value score (0, 1, 2 and 3, respectively). Medians (continuous) and proportions (categorical) of study variables were calculated using these ordinal values of the bacterial score. Predictors of the bacterial score, estimates, and 95% confidence intervals (CI) were derived from a proportional odds regression model. Potential predictors were included in the model based on prior studies of risk factors and comparing log-likelihoods of models.

The final model for the procedural variables was adjusted for time from bowel preparation to colonoscopy, use of split dosing, Boston bowel preparation scores, specific bowel preparations used, and study site.

The final model for the demographic/lifestyle factors was adjusted for time from bowel preparation, Boston bowel preparation score, study site, age (continuous), sex, BMI (underweight [BMI <18 kg/m^2^], nonobese [BMI 18–30] and obese [BMI >30 kg/m^2^]), physical activity (none, moderate, or vigorous), cigarette use pack-years (0 years, > 0–5 years, >5–10 years, >10 years), aspirin use within the last two weeks (yes/no), diabetes mellitus, hyperlipidemia, and alcohol use (≥ weekly, < weekly, or rarely/never use [<1 serving per month, 2 oz hard alcohol, 5 oz of wine or 8 oz of beer]). Because similar associations were observed for < weekly and rarely/never use alcohol, these two categories were combined for subsequent analyses.

The association between bacterial score and polyp risk was analyzed by multinomial logistic regression, stratifying by polyp type. Individuals missing polyp data were excluded from this analysis (N = 49, Figure 1a). Confounding risk factors were included in the final model if they were established risk factors and potential confounders: sex, age, cigarette use (current, former, never), BMI categories (<18 kg/m^2^, >18–30, >30), prior colon polyp (yes/no/don’t know), history of cholecystectomy (yes/no), diabetes mellitus (yes/no), hyperlipidemia (yes/no), alcohol use (less than weekly/at least weekly) and moderate or vigorous physical exercise (yes/no). Tests for trends were derived by including the categorical variable as a continuous factor in the model. Statistical analyses were completed using R studio (version 4.2.2). *p* values of ≤ 0.05 (2-sided probability) were considered statistically significant in all analyses.

## Results

### Procedural variables affecting biofilm detection

We first analyzed the colonic biofilm status of individuals undergoing screening colonoscopy without a history of or current CRC diagnosis. We utilized a semi-quantitative bacterial scoring system on a scale from 0–3 (Figure 1a,b). Data analyses after screening the first 100 individuals revealed a pronounced heterogeneity amongst biofilm prevalence at the three study sites: the Reading site (RE) had no detectable biofilms in their first 100 individuals, whereas the Green Spring (GS) and White Marsh (WM) sites had 16 (16%) and 12 (12%), respectively (Fisher’s exact *t*-test: RE vs. GS *p* < 0.0001; RE vs. WM *p* = 0.0003). To examine potential procedural differences between study sites, we thereafter normalized procedures to every extent possible, including switching to large capture biopsy forceps at RE, prescription of predominantly Miralax/Dulcolax or Suprep bowel preparations, and at least 5 h from bowel preparation completion to colonoscopy start time. Additionally, for a subset of individuals at each study site we examined biofilm status on 12 methacarn-fixed biopsies instead of 6.

We evaluated the impact of these changes on bacterial scores using a multivariable proportional odds logistic regression model (Figure 1c, Table S3). In the final adjusted model, increased time from bowel preparation completion (adjusted odds ratio [aOR] 1.05, 95% confidence interval [CI]: 1.00, 1.09), single prep dosing (aOR 1.48, 95% CI: 1.00, 2.19), and higher number of biopsies examined (12 vs. 6, aOR 2.34, 95% CI: 1.54, 3.55) were associated with increased bacterial scores. Patients from RE (aOR 0.16, 95% CI: 0.09, 0.29) and Suprep bowel preparation (aOR 0.40, 95% CI: 0.21, 0.75) were associated with lower bacterial scores.

### Biofilm distribution along the colon

**Figure 2.**
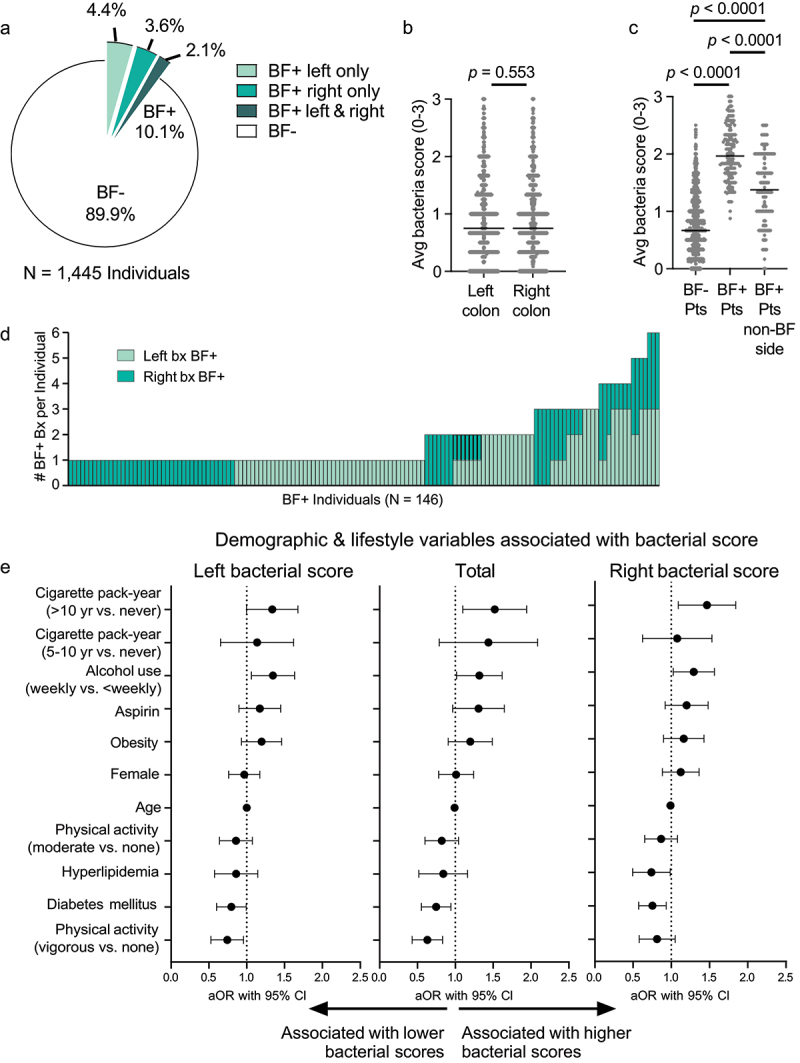
Distribution, demographic, and lifestyle features of bacterial biofilms. (a) Pie chart of biofilm detection rates on the left colon only (mint), right colon only (turquoise), or both colon sides (dark green) from 1,445 individuals with 6 biopsies obtained with large forceps. (b) Average bacterial scores on right vs. left colon. Mann-Whitney *p*-value. (c) Average total bacterial scores of biofilm-positive (BF+) individuals, biofilm-negative (BF-) individuals, and the non-biofilm side of BF+ individuals. Mann-Whitney *p*-values are shown. (d) Stacked bar chart of the number of biopsies (bx) per individual scored as BF+ either on the right (turquoise) or left (mint) colon. Each bar represents an individual. (e) Forest plots of adjusted proportional odds logistic regression model (aOR) of demographic and lifestyle factors associated with bacterial score, adjusted for procedural and demographic risk factors (see methods, Table 2). aOR with 95% CI are shown for the left colon, total colon, and right colon bacterial scores.

Given the pronounced effect of biopsy size, aggressive bowel preparation, and time from preparation on the biofilm status, we examined the distribution of biofilms along the gastrointestinal tract using a subcohort of 1,445 individuals with no history of or current CRC and for whom six biopsies for biofilm screening were collected with large forceps (Figures 1a, 2a–d). A total of 146/1,445 individuals (10.1%) harbored a biofilm on at least one of their colonic biopsies (Figure 2a). Biofilm rates on the left vs. right colon were not statistically different in healthy individuals (Figure 2a, 6.5% vs. 5.7%, respectively, Fisher’s exact t-test *p* = 0.393), in contrast to CRC where right-sided biofilms are much more prevalent.^1,2^ Average bacterial scores were also not significantly different between left and right sides of the colon (Figure 2b, *p* = 0.553). Few individuals had biofilms on both sides of their colon (Figure 2a, 2.1%; *N* = 30). However, in individuals who only had a biofilm on one side of their colon, the non-biofilm side had significantly higher bacterial scores compared to biofilm-negative individuals (Figure 2c, *p* < 0.0001). Additionally, 40% of individuals who were biofilm positive displayed biofilms on more than one of their six biopsies (Figure 2d). Three individuals were biofilm positive on all 6 biopsies (Figure 2d). These data suggest that while biofilms are largely focal and/or random in healthy individuals, a potential ‘field effect’ exists wherein higher bacterial scores in one region of the colon predisposes it to increased bacterial invasion elsewhere on the colon.

### Demographic and lifestyle variables associated with biofilms

We next examined the demographic and lifestyle variables associated with bacterial scores and biofilm detection. For this analysis, we excluded the non-optimal biopsies at RE that had been captured with the smaller (medium) forceps and were associated with shorter times from bowel preparation completion (<5 h) (see study Flowchart in Figure 1a). Individuals with a bacterial score of 3 were more likely to be male, smoked for greater than 10 years, used aspirin, drank more than weekly, were enrolled in the Green Spring study site, and had a longer time from completion of bowel prep to the start of colonoscopy (Table 1). Individuals with a bacterial score of 0 were more likely to have hyperlipidemia, be enrolled at the Reading study site, and had a shorter time from completion of bowel preparation to the start of colonoscopy (Table 1).

**Table 1.** Demographic characteristics of study subjects at enrollment across ordinally transformed values of bacterial scores (*N* = 1,605 individuals).

	Ordinal values of binned bacterial scores^1^				
Characteristic	0, *N* = 66	1, *N* = 888^*2*^	2, *N* = 546^*2*^	3, *N* = 105^*2*^	*p*-value^*3*^
Age^2^	62 (54, 69)	61 (54, 68)	61 (53, 67)	61 (56, 70)	.4
Female	36 (55%)	525 (59%)	282 (52%)	55 (52%)	.04
BMI^2^	27.8 (25.5, 32.9)	28.3 (24.7, 32.3)	28.7 (25.1, 33.0)	27.3 (23.5, 31.6)	.2
Diabetes mellitus	18 (27%)	212 (24%)	116 (21%)	22 (21%)	.5
Hyperlipidemia	16 (24%)	138 (16%)	37 (6.8%)	3 (2.9%)	<.001
Medication^4^					
Antibiotics	28 (42%)	400 (45%)	241 (44%)	46 (44%)	.6
Aspirin	24 (36%)	251 (28%)	191 (35%)	41 (39%)	.006
Insulin	1 (1.5%)	24 (2.7%)	16 (3.0%)	3 (3.0%)	>.9
Metformin	7 (11%)	83 (9.4%)	53 (9.9%)	10 (9.9%)	>.9
NSAIDs	34 (52%)	388 (44%)	273 (50%)	56 (53%)	.02
Health Center					<.001
Green Spring	6 (9.1%)	241 (27%)	268 (49%)	67 (64%)	
Reading	46 (70%)	375 (42%)	117 (21%)	4 (3.8%)	
White Marsh	14 (21%)	272 (31%)	161 (29%)	34 (32%)	
Time since prep^2^	10.8 (6.7, 13.6)	11.9 (8.9, 14.2)	13.2 (10.7, 15.7)	16.1 (12.4, 18.6)	<.001
Activity					.4
Vigorous	21 (32%)	251 (28%)	176 (32%	34 (32%)	
Moderate	35 (53%)	426 (48%)	228 (42%)	48 (46%)	
None	10 (15%)	208 (23%)	140 (26%)	23 (22%)	
Cigarette use					.1
Current	4 (6.1%)	95 (11%)	60 (11%)	12 (11%)	
Former	22 (33%)	271 (31%)	180 (33%)	47 (45%)	
Never	40 (61%)	522 (59%)	305 (56%)	46 (44%)	
Cigarette pack-year					.3
0 years	46 (70%)	562 (63%)	333 (61%)	51 (49%)	
>0–5 years	5 (7.6%)	84 (9.5%)	56 (10%)	14 (13%)	
>5–10 years	2 (3.0%)	56 (6.3%)	34 (6.2%)	8 (7.6%)	
>10 years	13 (20%)	186 (21%)	123 (23%)	32 (30%)	
Alcohol use	38 (58%)	437 (50%)	314 (60%)	58 (56%)	.06
History of polyps	31 (47%)	428 (48%)	257 (47%)	54 (51%)	
History of colon cancer	1 (1.5%)	13 (1.5%)	13 (2.4%)	1 (1.0%)	.6
Polyp type					.2
No polyp	32 (48%)	463 (52%)	289 (53%)	49 (47%)	
Hyperplastic	6 (9.1%)	61 (6.9%)	35 (6.4%)	7 (6.7%)	
Adenomatous	19 (29%)	287 (32%)	180 (33%)	38 (36%)	
Sessile serrated	6 (9.1%)	43 (4.8%)	17 (3.1%)	6 (5.7%)	
Synchronous	3 (4.5%)	27 (3.0%)	20 (3.7%)	1 (1.0%)	
Adenocarcinoma	0 (0%)	0 (0%)	3 (0.5%)	2 (1.9%)	

^1^Average bacterial scores across individuals were binned (0, > 0 to < 1, ≥1 and < 2, and ≥ 2) and then transformed into ordinal values (0, 1, 2, and 3, respectively; see Methods).

^2^Median (IQR); n (%).

^3^Kruskal-Wallis rank sum test for continuous variables; Fisher’s exact test for categorical variables.

^4^Antibiotics exposure was queried over the last 12 months. Aspirin and NSAID exposure was queried as days/week and tablets/week. Insulin and metformin were queried as yes/no.

We did not see any association between bacterial score and diet (Table S4). Significant variables were incorporated into a multivariable proportional odds regression model, along with other known influencers of colon cancer (Table 2, see Methods). Both unadjusted odds ratios (OR) and adjusted odds ratios (aOR) are shown in Table 2; Figure 2e shows forest plots of the aOR. For every one-unit increase in one of these variables, there is a proportional increase in the odds of a higher bacterial score reflected by the value of the coefficient.

**Table 2.** Estimates and 95% confidence intervals for demographic and lifestyle variables from proportional odds model of bacterial score and risk factors.

Characteristic	OR (95% CI)^1^	*p*-value	aOR (95% CI)^2^	*p*-value
Time from preparation	1.15 (1.12, 1.18)	<0.001	1.11 (1.08, 1.15)	<0.001
Boston Bowel preparation score	0.78 (0.72, 0.85)	<0.001	0.73 (0.66, 0.79)	<0.001
Health Center				
Green Spring	Ref		Ref	
Reading	2.59 (2.01, 3.36)	<0.001	3.80 (2.74, 5.26)	<0.001
White Marsh	5.14 (4.01, 6.61)	<0.001	4.84 (3.46, 6.77)	<0.001
Age	1.00 (0.99, 1.01)	0.9	0.99 (0.98, 1.00)	0.2
Female	0.78 (0.65, 0.95)	0.01	0.99 (0.79, 1.25)	>0.9
Cigarette pack-year				
0 years	Ref		Ref	
>0–5 years	1.31 (0.95, 1.81)	0.10	1.43 (0.99, 2.08)	0.06
>5–10 years	1.24 (0.83, 1.84)	0.29	1.34 (0.84, 2.13)	0.7
>10 years	1.29 (1.02, 1.63)	0.03	1.48 (1.12, 1.96)	0.01
Alcohol use				
Never/Less than Weekly	Ref		Ref	
Weekly	1.30 (1.07, 1.58)	0.01	1.29 (1.03, 1.63)	0.03
Aspirin	1.31 (1.07, 1.61)	0.009	1.28 (0.98, 1.66)	0.06
Hyperlipidemia	0.35 (0.25, 0.48)	<0.001	0.80 (0.54, 1.18)	0.3
Diabetes mellitus	0.84 (0.67, 1.06)	0.1	0.73 (0.56, 0.95)	0.02
Obesity	1.03 (0.85, 1.25)	0.8	1.17 (0.92, 1.50)	0.2
Physical activity				
None	Ref		Ref	
Moderate	0.80 (0.63, 1.02)	0.9	0.80 (0.61, 1.05)	0.1
Vigorous	1.01 (0.78, 1.30)	0.9	0.61 (0.44, 0.84)	0.002

^1^Unadjusted odds ratio (OR) based on univariable proportional odds logistic regression.

^2^Adjusted OR (aOR) from multivariable proportional odds logistic regression model adjusted for time from bowel preparation, Boston bowel preparation score, age, sex, study site, obesity, physical activity, cigarette use pack-years, aspirin use, diabetes mellitus diagnosis, hyperlipidemia, and alcohol use.

Lifestyle factors significantly associated with increased odds of higher bacterial score in the adjusted model included > weekly alcohol use compared to < weekly or no alcohol use (aOR 1.29, 95% CI: 1.03, 1.63) and greater than 10 pack-years of cigarettes compared to nonsmokers (aOR 1.48, 95% CI: 1.12, 1.96) (Figure 2e, Table 2). A trend toward higher bacterial score was detected for obesity (aOR 1.17, 95% CI: 0.92, 1.50; *p* < 0.2) (Figure 2e, Table 2). Conversely, factors associated with decreased odds of higher bacterial score included self- reported moderate physical activity (aOR 0.80, 95% CI: 0.61, 1.05), vigorous physical activity (aOR 0.61, 95% CI: 0.44, 0.84), and diagnosis of diabetes mellitus (aOR 0.73, 95% CI: 0.56, 0.95). These variables were equally associated with both left and right-sided biofilms (Figure 2e). However, lifestyle factors contributed to a much smaller percentage of the inter-individual variability in the model compared to procedural variables (Figure S1).

### Association of biofilms on normal tissues with polyp risk

**Figure 3.**
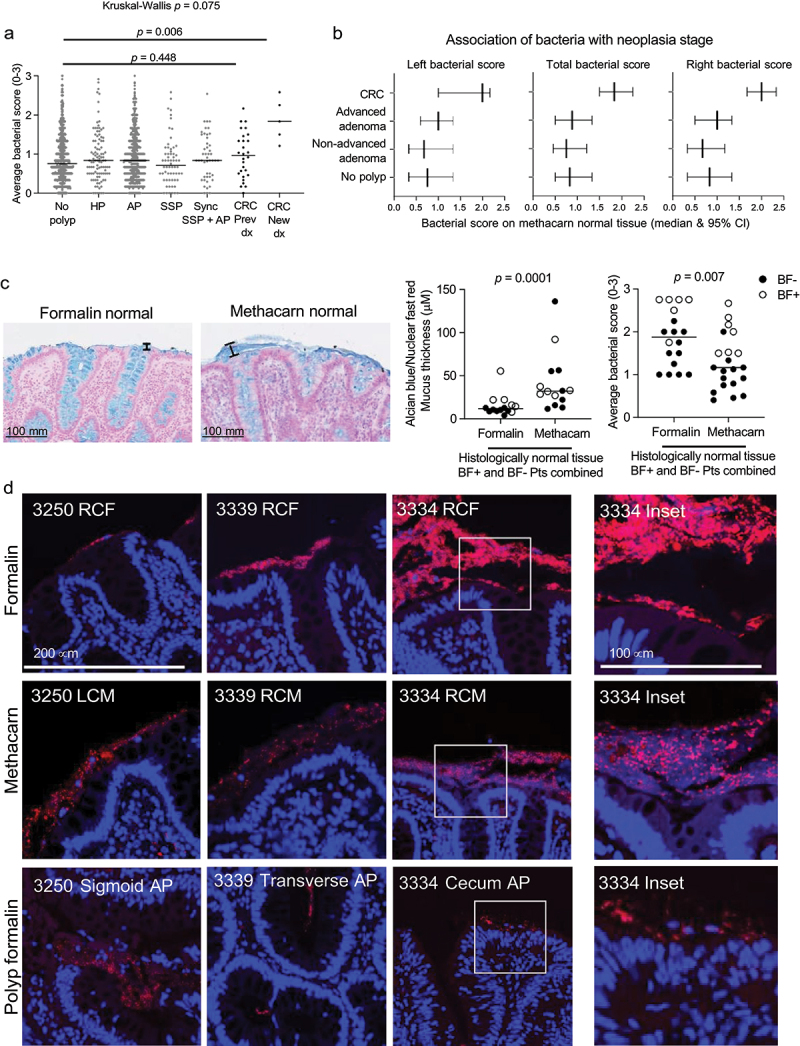
Association of biofilms on normal tissues with polyp status. (a) Bacterial scores on methacarn-fixed normal tissues from individuals with or without neoplasia (HP, AP, SSP, and CRC). Kruskal-Wallis and Mann-Whitney p-values are shown. (b) Bacterial scores on methacarn-fixed normal tissues from individuals stratified by neoplasia stage (CRC, advanced adenoma, non-advanced adenoma, or no polyp). (c) Left: representative Alcian blue mucus staining with peak mucus indicated in brackets. Middle: average mucus thickness. Right: average bacterial scores of FFPE vs. methacarn normal tissue pairs. Mann-Whitney p-values are shown. (d) FISH images from 3 BF+ individuals, including FFPE normal (top row), methacarn normal (middle row), and FFPE polyp (bottom row). Far right: inset of the samples. Blue = DAPI, red = EUB338. Images were acquired at 40X on a zeiss 780 confocal microscope. RCF, right colon formalin; LCM, left colon methacarn; AP, adenomatous polyp.

Given the association of numerous CRC risk factors with bacterial scores in our analyses above, we next examined whether bacterial scores on histologically normal tissues were associated with neoplasia. Individuals with polyps did not display significantly different bacterial scores on their normal tissue compared to polyp-free controls (Figure 3a). This was also confirmed by a multinomial logistic regression model (Table S5). Only individuals with newly diagnosed CRC displayed a significantly elevated bacterial score on their methacarn-fixed normal biopsies compared to polyp-free controls (Figure 3a, Mann-Whitney *p* = 0.006).

Importantly, all 5 of the corresponding CRCs from these individuals were located in the left (sigmoid or rectal) region of the colon, which we would anticipate to have even lower bacterial scores than the biofilm-rich proximal CRCs.^1,2^ Similarly, when we examined the impact of neoplasia stage on bacterial score, median total bacterial scores were significantly higher in individuals with CRC compared to advanced adenomas, and there was a trend toward higher bacterial scores in individuals with advanced adenomas compared to non-advanced adenomas or polyp-free controls (Figure 3b). These patterns were consistent across both left colon, right colon, and total bacterial scores (Figure 3b).

### Impact of fixative type on biofilm detection

These associations of bacteria on histologically normal tissues with lifestyle factors and potential polyp risk led us to ask whether an even stronger association might be detected on the polyp tissues themselves. However, polyps from pathology are preserved in FFPE, an aqueous fixative that can thin, dissolve, or strip away mucus alongside any potential mucosally associated bacteria.^3,18^ To assess the degree to which biofilm detection might be impacted by FFPE fixation, we compared mucus quality and bacterial scores from paired FFPE and methacarn-fixed normal tissues from a subset of 21 individuals from our cohort.

As expected, FFPE tissues displayed significantly thinner mucus than paired, methacarn- fixed tissues on both Alcian blue/Nuclear Fast Red stained tissues (Figure 3c, *p* = 0.0001 Wilcoxon paired t-test) and mucus autofluorescence (Figure S2A,B, *p* = 0.002 Wilcoxon paired t-test). However, FFPE bacterial scores were significantly higher than methacarn-fixed tissues from the same individuals (Figure 3c, *p* = 0.007 Wilcoxon paired t-test). Concordant biofilms could be observed on several individuals’ paired FFPE polyp, FFPE normal, and methacarn normal tissues (Figure 3d). Overall, these data suggested that FFPE is sufficient for analysis of highly adherent and/or invasive bacteria on normal tissue as well as polyps despite the loss or thinning of the outer mucus layer.

### Biofilms on polyp tissues

**Figure 4.**
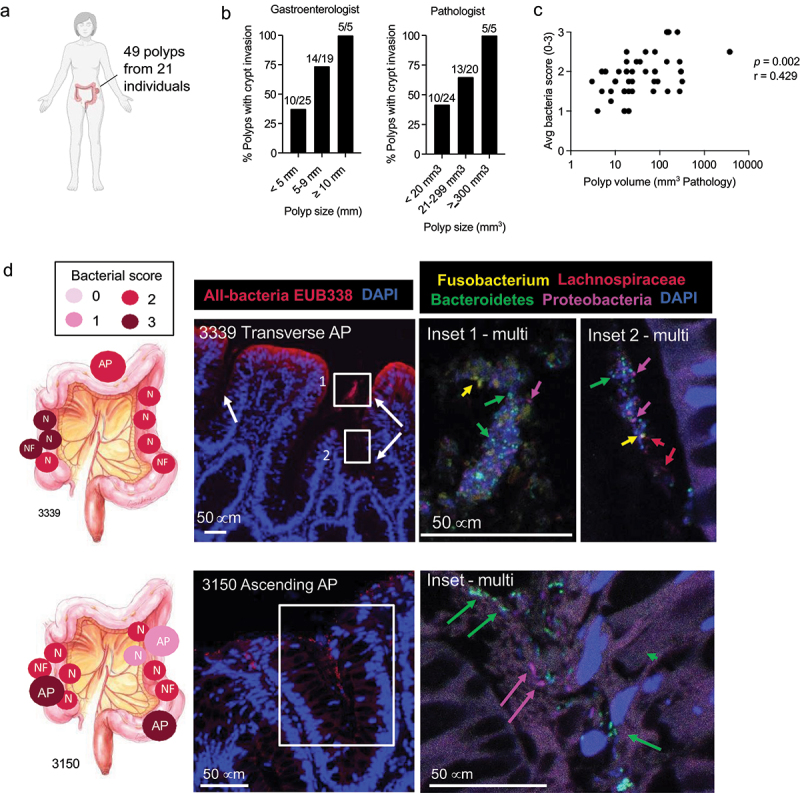
Polyps are associated with pronounced microbial crypt invasion. (a) Forty-nine FFPE polyps from 21 individuals (range: 1–5 polyps per individual) were screened for bacterial biofilms. (b) Prevalence of crypt invasion as a factor of polyp size, based on polyp diameter estimates (mm) by the scoping gastroenterologist or volumetric (mm^3^) measurements recorded by the pathologist. (c) Spearman’s rank correlation of polyp bacterial score vs. polyp volume (*N* = 49 polyps). (d) Left panel: representative examples of biofilm and polyp biogeography on normal methacarn-fixed tissues (N), normal FFPE tissues (NF), and FFPE adenomatous polyps (AP) on two individuals. Middle panel: FISH with universal probe EUB338 and DAPI counterstain. Right panel: multi-probe FISH inset of the polyps with probes targeting bacteroidetes/*Prevotella* (green), Lachnospiraceae (red), Proteobacteria (magenta), and *Fusobacterium* (yellow), with DAPI counterstain (blue). Arrows denote examples of positive staining for each bacteria type. Images were acquired on a zeiss 780 confocal at 40X with lambda scanning.

We next analyzed the polyp bacteria of 49 FFPE polyps from the above 21 individuals (Figure 4a). This included 7 biofilm-positive (Figure S3) and 14 biofilm-negative individuals (Figure S4) based on scores from normal methacarn tissue. As individual polyps (and their resulting tumors) are genetically distinct, each polyp was analyzed as a separate entity. ^19–21^ Three of the 49 polyps were biofilm positive: one was a sigmoid AP from individual 3339 whose normal mucosa was also biofilm positive (Figure 4d), and two (ascending AP and sigmoid AP) were from individual 3150 whose normal tissues were biofilm negative (Figure 4d). No differences were observed in on-polyp bacterial scores between AP (*N* = 34), SSP (*N* = 11), and HP (*N* = 8) (Figure S5A, Kruskal-Wallis *p* = 0.319). However, bacterial scores from FFPE tissues from the biofilm-positive individuals (classified by methacarn biofilm status) were all significantly higher than biofilm-negative individuals (Figure S5B, *p* = 0.020 FFPE polyps; *p* = 0.006 FFPE normal; Mann-Whitney *p*-values). These data further support our findings that while biofilm status varies across the colonic axis, biofilms present even on only one colon side associate with higher bacterial scores elsewhere in the colon (Figure 2c).

Finally, although bacterial scores on the polyps were not significantly different from paired normal FFPE tissues (Figure S5B), polyps exhibited a marked shift in bacterial localization, with prominent crypt invasion regardless of biofilm status (Figure 4b). This was particularly evident in larger polyps (≥10 mm in diameter or a volume ≥300 mm^3^) (Figure 4b).

Indeed, bacterial scores significantly correlated with polyp size, regardless of whether each polyp was analyzed separately (Figure 4c, *p* = 0.002, *r* = 0.429 Spearmans’ rank correlation) or as an average per person for individuals who had more than one polyp (Figure S5C, *p* = 0.003, *r* = 0.609 Spearman's rank correlation). Finally, at the composition level by multi-probe FISH, both luminal and crypt-invasive aggregates in all polyps were polymicrobial, consisting predominantly of Bacteroidetes/*Prevotella* spp. and, to a lesser extent, Lachnospiraceae and Proteobacteria (Figure 4d). *Fusobacterium* spp. were rare, and never in clusters or microcolonies as previously observed in CRC tissues (Figure 4d).^1,2,4^

## Discussion

Bacteria stressed by harsh environments form protective biofilms, but no study to date has investigated whether biofilms in the colon have analogous, host-associated environmental risk factors. Our study, encompassing nearly 13,000 biopsies and 49 polyps from 2,051 screening colonoscopy individuals, sought to advance our understanding of the complex ecology and associated risk factors in the colon. On normal tissues (“off-polyp”), we found that greater than 10 pack-years of smoking and weekly alcohol use associated with increased biofilm prevalence, whereas moderate or vigorous physical activity and diabetes were associated with reduced biofilm prevalence in a screening colonoscopy cohort. There was also a non-significant, positive association between obesity and biofilms. No significant association was found between biofilm presence and adenomas, but we did note a positive association between bacterial score and adenocarcinoma diagnosis as documented in prior clinical studies,^1,2^ as well as a trend toward increased bacterial presence in individuals with advanced adenomas compared to non- advanced adenomas. Additionally, “on-polyp” FISH analyses demonstrated more crypt invasion as polyps advanced in size. Together, these data support a co-evolution of bacterial biofilm development along the adenoma-carcinoma sequence.

A majority of the biofilm risk factors that we identified are also established risk factors of CRC, with plausible linkages to biofilms.^22^ For example, although no prior dataset has examined the risk of smoking on colonic biofilms, oral biofilms have been demonstrated in people who smoke cigarettes and are associated with inflammatory mucosal changes.^23^ Furthermore, accumulating research documents a link between current smokers and changes in stool microbiome taxa, alterations in mucin composition, colonic mucosal inflammatory response, and reactive oxygen species.^24^ Similarly, alcohol consumption has been correlated with changes in the microbiome that leads to increased intestinal mucosal inflammation and permeability, causing endotoxemia, systemic inflammation, and colonic tissue damage.^25^ Besides obesity and alcohol consumption, our study did not reveal any additional association between diet and biofilms. This could be due to the fact that our limited dietary questionnaire was unable to parse out more granular associations, such as the contribution of different types of alcohol, different types of meat or grains, etc. Additionally, individuals enrolled in our study were prescribed a standard, low-residue diet for at least 2–3 days prior to colonoscopy (Table S1), which may have confounded potential dietary associations with biofilm status. Nevertheless, the association of alcohol consumption with biofilms, and trend toward obesity with biofilms, suggests that a potential linkage between diet, alcohol consumption, and biofilms should be assessed thoroughly in future studies. While our initial study was focused on the epidemiology of bacterial biofilms, assessment of either focal or systemic inflammation (e.g., tissue staining or fecal calprotectin) in future studies in parallel with biofilm and dietary assessments would further help to inform the pathophysiology of colonic biofilms. Importantly, our prior studies documented that biofilms on normal colonic biopsies in individuals undergoing colonoscopy displayed epithelial changes in IL-6, consistent with colonic biofilms inducing shifts in mucosal inflammation.^1^

We also found that moderate or vigorous physical activity predicted decreased bacterial scores. There was evidence of a dose effect, with a stronger association seen in vigorous versus moderate physical activity. Prior cross-sectional and interventional studies have shown that higher levels of physical activity and cardiorespiratory fitness are associated with beneficial microbial changes including increased short-chain fatty acid (SCFA) producers and elevated fecal SCFA.^26,27^ By acting to increase bacterial diversity and SCFA-producing species, exercise may promote the integrity of the gut mucosal layer and reduce bacterial biofilm presence.^28^

Not all CRC-associated variables tracked with biofilm status, however. For example, while both obesity and type 2 diabetes are linked to CRC,^29,30^ obesity trended toward a positive association with bacterial score, while diabetes was associated with reduced bacterial score in our study. While our questionnaire did not differentiate between different types of diabetes mellitus, the vast majority of cases are likely to be type 2 based on current epidemiology in the US. Nevertheless, inclusion of type 1 diabetes mellitus may have confounded this analysis. More plausible, however, is that the use of anti-diabetic medications may be confounding the relationship between biofilms and diabetes. Metformin, in particular, has been shown to have substantial impacts on the gut microbiota that in turn may prevent or disrupt mucus-invasive biofilm formation. These include direct antimicrobial activity,^31^ adjuvant anti-microbial activity ^32–34^ and inhibition of quorum sensing, a key signal required for biofilm formation.^35,36^ Metformin’s effects on the host that may also reduce or prevent biofilm formation include thickening of the protective mucus layer in the gut and immunomodulatory activities on gut immune cells.^37^

Age was also not associated with biofilm status in our study, despite advanced age being strongly linked to CRC. Notably, endoscopically visible biofilms were not associated with age in the Baumgartner study, either, despite otherwise strong associations between biofilms and IBS and UC disease states.^6^ It is possible that a lack of association between biofilms and age is part of the broader shift in the age distribution of CRC, with increasing rates in younger individuals (so-called early-onset CRC, or EO-CRC). However, only 11% of the individuals in our screening colonoscopy cohort were <50 years old. Further studies will be necessary to parse out these complex questions. Nuances such as these support the hypothesis that while most invasive biofilms in the colon are likely detrimental and are associated with other CRC risk factors, they are nevertheless part of a complex intermediate step in cancer development that do not always correctly predict polyp status at a given time point.

Importantly, we also identified a number of procedural factors that may influence biofilm detection. Rapid changes in clinical practice over the course of our study allowed us to examine these factors in detail. Most notably, bacterial scores were inversely proportional to the quality of the bowel preparation. More rigorous preparations, such as shorter time frames from completion of the bowel preparation to colonoscopy and use of split preps, led to significantly lower bacterial scores. Significant differences were also observed with biopsy forceps size, study site, and scoping physician, with modest effects by bowel preparation type. These differences suggest that even in individuals with less stringent bowel preparations, the natural biofilm state is still likely perturbed. This represents an important caveat of our study. Development of animal models for *in vivo* colonic biofilms and/or assessment of FFPE blocks from individuals prior to the use of modern-day colonoscopy preparations may be necessary to confirm the true epidemiology of *in situ* biofilms. Prospectively controlling for these procedural variables will be critical for future studies of not only colonic biofilms but also potentially any tissue-based colonic microbiome study and represents an important finding from our study.

The overall biofilm prevalence in our study (10.1%) was lower than a previous cohort of healthy individuals from Johns Hopkins Hospital^1^ and a recent Netherlands cohort (50%),^5^ but similar to endoscopically visible biofilms in control subjects from Austria and Germany (6%).^6^ Potential differences between the present study and the Netherlands cohort include differences in bowel preparation as well as a less stringent biofilm definition (100 µm rather than 200 µm) in the Netherlands study. Nonetheless, the results herein confirm that microscopic biofilms are not inherently predisposed to form on one side of the colon or the other in healthy individuals.^1,5^

While biofilms on histologically normal tissues were not a biomarker or predictor of colonic polyp status at the current colonoscopy, bacterial scores and crypt invasion analyzed directly on the polyp tissues strongly correlated with increasing polyp size, further supporting that polyps and the tumor microbial environment co-evolve. These highly invasive bacterial aggregates on polyp tissues mimicked the prolific invasion observed in CRC tissues where not only crypt but deep tumor invasion are seen.^1,2,4^

Finally, our comparison of different fixatives (FFPE vs. methacarn) revealed that although mucus was better preserved by methacarn, the FFPE scores for adherent or invasive bacteria within 1 µm of the epithelium were comparable to methacarn bacterial scores. These data suggest that FFPE is suitable for analysis of highly adherent bacterial communities, despite its disadvantages in resolving mucus quality and/or outer mucus microbial communities. Given our prior data that shifts in clinical practice, in particular the use of pre-operative oral antibiotics and/or rigorous bowel preparations for CRC resections, may severely – if temporarily – deplete the *in situ* microbial community,^4^ utilization of retrospective FFPE cohorts may become increasingly important in the elucidation of microbial factors associated with CRC.

Limitations of our study include the aforementioned variations in procedural factors and limited dietary information as well as a largely non-diverse patient population. These data highlight both the challenges of detecting biofilms in screening colonoscopy cohorts and also provide the first in-depth analysis of procedural, demographic, and lifestyle factors that impact biofilm prevalence in the colon. Our observations in a limited polyp cohort contribute to our understanding of the early bacterial:polyp transitions, and our accessible scoring system for mucus-invasive bacteria may enable cross-cohort comparisons in the future.

## Supplementary Material

SUPPLEMENTARY MATERIAL.docx

## Data Availability

Deidentified data will be deposited in the Johns Hopkins Research Data Repository. Additional details are available upon request.
