# Supplementary material for: Epidemiology of bacterial biofilms on polyps and normal tissues in a screening colonoscopy cohort
Source: Gut Microbes. 2025 Jan 18;17(1):2452233. doi: 10.1080/19490976.2025.2452233 (PMC12716056; doi:10.1080/19490976.2025.2452233)
Supplement: SUPPLEMENTARY MATERIAL.docx [file KGMI_A_2452233_SM6569.docx]

**SUPPLEMENTARY MATERIAL**

**Supplementary Figure 1. Contribution of procedural, demographic, and lifestyle variables to the multivariable proportional odds regression model.** (**A**) Percent variance (inter-individual variability) from adjusted R^2^ values of the multivariable proportional odds regression model explained by procedural and demographic/lifestyle factors. Incorporation of demographic/lifestyle features into the model only slightly improved the percent variance controlled for by the model, which was focused primarily on procedural factors.

**Supplementary Figure 2. Effect of fixative type on mucus quality**. (**A**) Semi-quantitative scoring of mucus (scale 0-2, 2 being excellent mucus, see Methods) on FISH-stained slides, using autofluorescence of the mucus to visualize. Samples were derived from the same subset of 21 screening colonoscopy cohort individuals utilized in Fig 3 (N = 7 biofilm positive, N = 14 biofilm negative individuals). Wilcoxon paired t-tests are shown for intra-individual pair-wise comparisons of FFPE vs. methacarn.

**Supplementary Figure 3. Distribution of bacterial scores along the colonic axis in biofilm-positive individuals (N=7).** (**A-B**) Biogeography of bacterial scores on methacarn-fixed normal tissues (N), FFPE normal tissues (NF), and FFPE polyps from biofilm-positive individuals (BF+, as determined on methacarn tissue) with (**A**) AP only or (**B**) both AP and SSP.

**Supplementary Figure 4. Distribution of bacterial scores along the colonic axis in biofilm-negative individuals (N=14).** (**A-B**) Biogeography of bacterial scores on methacarn-fixed normal tissues (N), FFPE normal tissues (NF), and FFPE polyps from biofilm-negative individuals (BF-, as determined on methacarn tissue) with (**A**) AP only or (**B**) both AP and SSP.

**
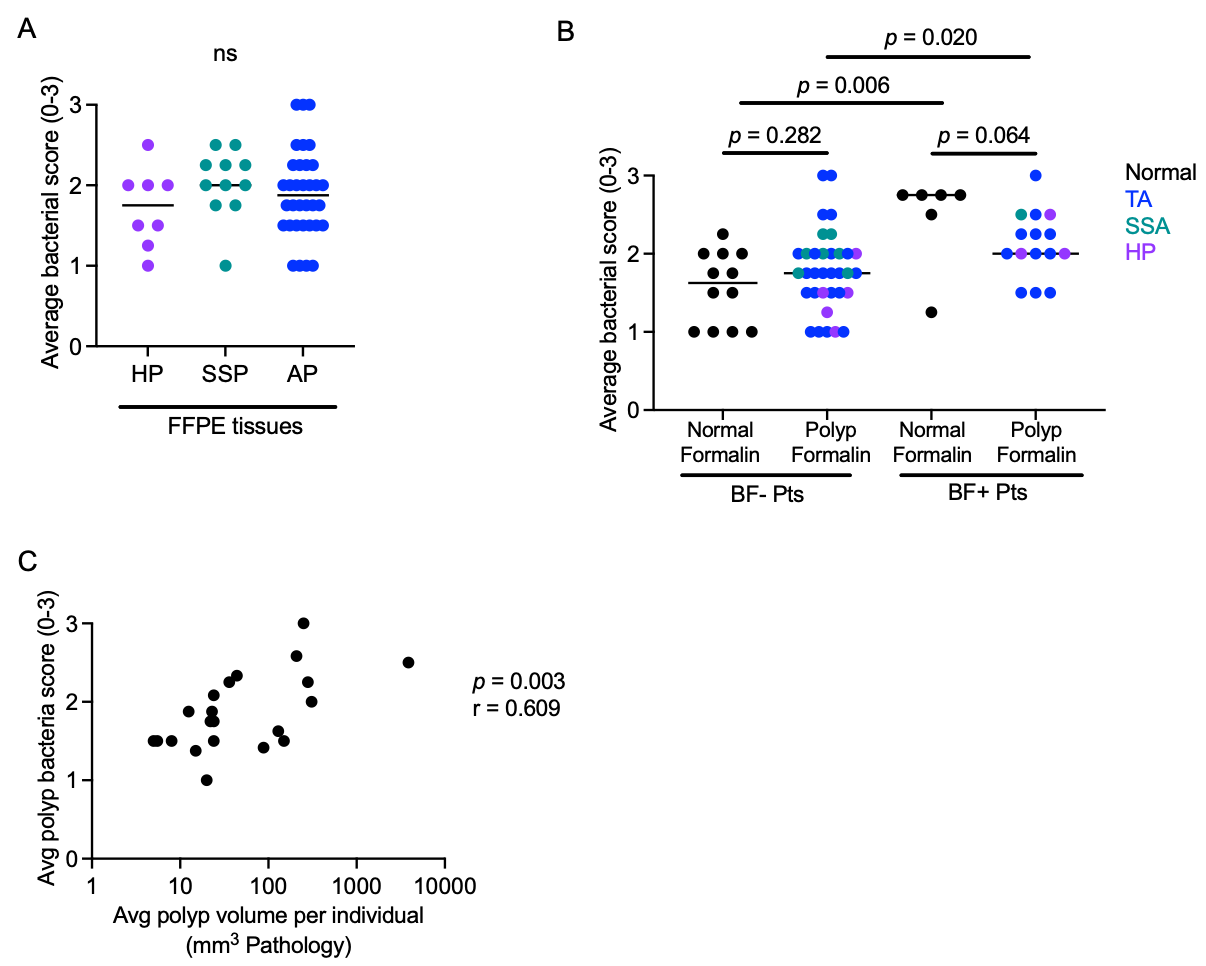
**

**Supplementary Figure 5. Bacterial FISH on polyp tissues compared to paired normal.** (**A**) Average bacterial scores on 49 polyp FFPE tissues from 21 individuals, segregated by polyp type (HP = hyperplastic, SSP = sessile serrated, AP = adenomatous). Each dot represents a single polyp. (**B**) Paired polyp and normal FFPE tissues from biofilm-positive and biofilm-negative individuals. Wilcoxon *p*-values are shown for paired data from the same patient (polyp vs. paired normal); Mann-Whitney *p*-values are shown for biofilm-positive vs. biofilm-negative comparisons between different patients. (**C**) Spearman’s correlation of average bacterial score on polyp tissues vs. average polyp volume from pathology measurements (mm^3^). Each dot represents the average value for each individual patient; for patients with multiple polyps, bacterial scores and polyp volumes were averaged to create a single value.

**Supplementary Table 1. Bowel preparation formulations by study site and physician**

**Supplementary Table 2. FISH probes**

**Supplementary Table 3. Multivariate model of procedural factors influencing bacterial score in biopsies acquired with large biopsy forceps**

| **Characteristic** | **OR (95% CI)^1^** | **p-value** | **aOR (95% CI)^2^** | **p-value** |
| --- | --- | --- | --- | --- |
| Time from preparation | 1.15 (1.12, 1.18) | <0.001 | 1.05 (1.00, 1.09) | 0.05 |
| Split vs single prep dosing |  |  |  |  |
| Split | — |  | — |  |
| Single | 2.40 (1.87, 3.09) | <0.001 | 1.48 (1.00, 2.19) | 0.04 |
| Boston bowel prep score | 0.78 (0.72, 0.85) | <0.001 | 0.71 (0.65, 0.78) | <0.001 |
| Endoscopy study site |  |  |  |  |
| Green Spring | — |  | — |  |
| Reading | 0.19 (0.15, 0.25) | <0.001 | 0.16 (0.09, 0.29) | <0.001 |
| White Marsh | 0.50 (0.40, 0.64) | <0.001 | 0.13 (0.00, 12.2) | 0.4 |
| Bowel prep type |  |  |  |  |
| Miralax/Dulc | — |  | — |  |
| Miralax/Dulc/MagCit | 0.34 (0.09, 1.15) | 0.09 | 0.44 (0.11, 1.80) | 0.3 |
| Miralax/Dulc/MoM | 0.86 (0.49, 1.49) | 0.6 | 0.83 (0.44, 1.5) | 0.6 |
| MagCit/Dulc | 0.24 (0.03, 1.64) | 0.2 | 0.13 (0.01, 1.63) | 0.1 |
| Golytely | 1.00 (0.70, 1.41) | 1.0 | 0.67 (0.41, 1.10) | 0.1 |
| Nulytely | 0.95 (0.44, 1.96) | 0.9 | 0.80 (0.33, 1.96) | 0.6 |
| Colytely | 1.04 (0.47, 2.20) | 0.9 | 1.88 (0.81, 4.34) | 0.1 |
| Moviprep | 1.37 (0.90, 2.09) | 0.1 | 1.48 (0.74, 2.97) | 0.3 |
| Suprep | 0.52 (0.32, 0.82) | 0.01 | 0.40 (0.21, 0.75) | 0.005 |
| Clenpiq | 0.96 (0.57, 1.59) | 0.9 | 1.45 (0.49, 4.32) | 0.5 |
| Physician |  |  |  |  |
| 1 | — |  | — |  |
| 2 | 2.47 (1.33, 4.62) | 0.004 | 0.40 (0.15, 1.07) | 0.07 |
| 3 | 7.59 (4.41, 13.3) | <0.001 | 1.19 (0.79, 1.79) | 0.4 |
| 4 | 1.21 (0.67, 2.19) | 0.5 | 1.02 (0.56, 1.87) | >0.9 |
| 5 | 1.58 (0.90, 2.82) | 0.1 | 3.98 (0.04, 360) | 0.5 |
| 6 | 0.85 (0.47, 1.55) | 0.6 | 0.80 (0.43, 1.46) | 0.5 |
| 8 | 2.37 (0.37, 13.1) | 0.3 | 1.16 (0.57, 2.37) | 0.4 |
| 9 | 1.28 (0.64, 2.55) | 0.5 | 11.1 (0.12, 993) | 0.7 |
| 10 | 4.19 (2.43, 7.33) | <0.001 | 0.77 (0.40, 1.46) | 0.3 |
| 11 | 0.64 (0.34, 1.22) | 0.2 | 5.19 (0.06, 477) | 0.4 |
| # of biopsies taken (12 vs 6) | 1.08 (0.76, 1.52) | 0.7 | 2.34 (1.54, 3.55) | <0.001 |
| ^1^ Unadjusted odds ratio (OR) based on univariable proportional odds logistic regression  ^2^ Adjusted OR (aOR) from multivariable proportional odds logistic regression model adjusted for time from preparation, bowel preparation score, study site, bowel preparation type, split vs single dosing, physician, and number of biopsies screened | | | | |

**Supplementary Table 4.** Lack of dietary association with biofilms in study subjects across ordinally transformed values of bacterial scores (N = 1,605 individuals)

|  | **Ordinal values of binned bacterial scores^1^** | | | | |
| --- | --- | --- | --- | --- | --- |
| **Characteristic** | **0**, N = 66*^2^* | **1**, N = 881*^2^* | **2**, N = 538*^2^* | **3**, N = 101*^2^* | **p-value***^3^* |
| Yogurt |  |  |  |  | 0.6 |
| I do not eat yogurt | 17 (26%) | 196 (22%) | 140 (26%) | 23 (23%) |  |
| I have eaten yogurt in the last year | 7 (11%) | 109 (12%) | 62 (12%) | 13 (13%) |  |
| Yogurt monthly | 18 (27%) | 185 (21%) | 110 (20%) | 16 (16%) |  |
| Yogurt weekly | 24 (36%) | 391 (44%) | 226 (42%) | 49 (49%) |  |
| Meat |  |  |  |  | 1.0 |
| I do not eat meat | 1 (1.5%) | 12 (1.4%) | 10 (1.9%) | 3 (3.0%) |  |
| I have eaten meat in the last year | 0 (0%) | 6 (0.7%) | 4 (0.7%) | 2 (2.0%) |  |
| Meat monthly | 1 (1.5%) | 31 (3.5%) | 20 (3.7%) | 2 (2.0%) |  |
| Meat weekly | 64 (97%) | 832 (94%) | 504 (94%) | 94 (93%) |  |
| Cheese |  |  |  |  | 0.3 |
| Cheese monthly | 5 (7.6%) | 99 (11%) | 74 (14%) | 16 (16%) |  |
| Cheese weekly | 58 (88%) | 741 (84%) | 436 (81%) | 79 (78%) |  |
| I do not eat cheese | 0 (0%) | 20 (2.3%) | 13 (2.4%) | 2 (2.0%) |  |
| I have eaten cheese in the last year | 3 (4.5%) | 20 (2.3%) | 14 (2.6%) | 4 (4.0%) |  |
| Missing | 0 (0%) | 1 (0.1%) | 1 (0.2%) | 0 (0%) |  |
| Milk |  |  |  |  | 0.3 |
| I do not eat milk | 28 (42%) | 293 (33%) | 179 (33%) | 30 (30%) |  |
| I have eaten milk in the last year | 10 (15%) | 78 (8.9%) | 37 (6.9%) | 7 (6.9%) |  |
| Milk monthly | 10 (15%) | 117 (13%) | 69 (13%) | 10 (9.9%) |  |
| Milk weekly | 18 (27%) | 392 (44%) | 252 (47%) | 54 (53%) |  |
| Missing | 0 (0%) | 1 (0.1%) | 1 (0.2%) | 0 (0%) |  |
| Fish |  |  |  |  | 0.2 |
| Fish monthly | 29 (44%) | 290 (33%) | 166 (31%) | 32 (32%) |  |
| Fish weekly | 31 (47%) | 518 (59%) | 324 (60%) | 62 (61%) |  |
| I do not eat fish | 5 (7.6%) | 32 (3.6%) | 19 (3.5%) | 2 (2.0%) |  |
| I have eaten fish in the last year | 1 (1.5%) | 40 (4.5%) | 28 (5.2%) | 5 (5.0%) |  |
| Missing | 0 (0%) | 1 (0.1%) | 1 (0.2%) | 0 (0%) |  |
| Egg |  |  |  |  | 0.6 |
| Egg monthly | 12 (18%) | 177 (20%) | 118 (22%) | 15 (15%) |  |
| Egg weekly | 52 (79%) | 655 (74%) | 388 (72%) | 80 (79%) |  |
| I do not eat egg | 0 (0%) | 17 (1.9%) | 15 (2.8%) | 3 (3.0%) |  |
| I have eaten egg in the last year | 2 (3.0%) | 30 (3.4%) | 17 (3.2%) | 3 (3.0%) |  |
| Missing | 0 (0%) | 2 (0.2%) | 0 (0%) | 0 (0%) |  |
| *^1^*Average bacterial scores across individuals were binned (0, > 0 to <1, >1 and <2, and >2) and then transformed into ordinal values (0, 1, 2, and 3, respectively; see Methods).  *^2^* Median (IQR); n (%)  *^3^* Kruskal-Wallis rank sum test for continuous variables; Pearson’s Chi-squared test; Fisher’s exact test for categorical variables | | | | | |

**Supplementary Table 5**. **Associations between demographic factors, bacterial scores, and polyp types using multinomial logistic regression**

| **Characteristic** | **aOR (95%CI)***^1^* | **aOR (95%CI)***^1^* | **aOR (95%CI)***^1^* | **aOR (95%CI)***^1^* | |
| --- | --- | --- | --- | --- | --- |
|  | **Hyperplastic** | **Adenomatous** | **Sessile serrated** | **Synchronous** | |
| Age | 0.99 (0.96, 1.01) | **1.03 (1.01, 1.05)** | 0.99 (0.96, 1.02) | **1.04 (1.00, 1.08)** | |
| Female | 1.08 (0.69, 1.69) | 0.84 (0.66, 1.08) | 1.08 (0.64, 1.83) | **0.52 (0.28, 0.97)** | |
| BMI |  |  |  |  | |
| Normal | Ref | Ref | Ref | Ref | |
| Obese | 1.20 (0.68, 2.12) | **1.69 (1.22, 2.34)** | 0.84 (0.44, 1.61) | 2.01 (0.84, 4.84) | |
| Overweight | 1.20 (0.69, 2.08) | **1.53 (1.11, 2.09)** | 0.75 (0.40, 1.42) | 1.97 (0.86, 4.50) | |
| Hyperlipidemia | 1.15 (0.49, 2.73) | 0.79 (0.53, 1.19) | 0.75 (0.32, 1.75) | 0.53 (0.19, 1.48) | |
| Bacterial score |  |  |  |  | |
| 0 | Ref | Ref | Ref | Ref | |
| 1 | 0.50 (0.19, 1.28) | 1.07 (0.57, 1.98) | 0.50 (0.19, 1.30) | 0.71 (0.19, 2.57) | |
| 2 | 0.42 (0.16, 1.13) | 0.98 (0.52, 1.87) | **0.31 (0.11, 0.90)** | 0.77 (0.20, 2.93) | |
| 3 | 0.36 (0.10, 1.30) | 1.10 (0.51, 2.38) | 0.61 (0.16, 2.29) | 0.20 (0.02, 2.20) | |
| Aspirin use | 0.74 (0.44, 1.24) | 1.15 (0.88, 1.50) | 1.13 (0.63, 2.03) | 0.96 (0.50, 1.86) | |
| Cigarette pack-year |  |  |  |  | |
| 0 years | Ref | Ref | Ref | Ref | |
| >0-5 years | 0.75 (0.33, 1.73) | 1.09 (0.73, 1.62) | **2.03 (1.02, 4.03)** | 1.33 (0.52, 3.40) | |
| >5-10 years | 0.85 (0.32, 2.23) | 1.04 (0.64, 1.71) | 1.40 (0.56, 3.50) | 1.50 (0.50, 4.53) | |
| >10 years | 1.52 (0.91, 2.54) | **1.46 (1.10, 1.95)** | 0.62 (0.28, 1.37) | 1.06 (0.50, 2.25) | |
| Diabetes mellitus | 0.80 (0.48, 1.34) | 1.04 (0.66, 1.15) | 0.89 **(**0.50, 1.59**)** | 0.71 (0.33, 1.51) | |
| Alcohol intake |  |  |  |  | |
| Less than weekly | Ref | Ref | Ref | Ref | |
| More than weekly | 0.84 (0.54, 1.30) | 1.04 (0.82, 1.32) | 0.98 (0.59, 1.64) | 1.49 (0.79, 2.80) | |
| History of polyps |  |  |  |  | |
| Never | Ref | Ref | Ref | Ref | |
| Yes | 1.65 (1.05, 2.59) | **1.86 (1.44, 2.39)** | **2.27 (1.32, 3.89)** | 1.65 (0.87, 3.12) | |
| Don’t know | 0.70 (0.16, 3.06) | 1.72 (0.94, 3.13) | 1.10 (0.24, 4.98) | 1.45 (0.31, 6.78) | |
| Study site |  |  |  |  | |
| Green Spring | Ref | Ref | Ref | Ref | |
| Reading | **0.44 (0.23, 0.86)** | 0.92 (0.66, 1.29) | 1.05 (0.57, 2.10) | 1.13 (0.52, 2.46) | |
| White Marsh | 0.67 (0.39, 1.15) | 0.76 (0.56, 1.04) | 0.52 (0.25, 1.09) | 0.69 (0.31, 1.5) | |
| Physical activity |  |  |  |  | |
| None | Ref | Ref | Ref | Ref | |
| Moderate | 0.75 (0.44, 1.28) | 1.10 (0.82, 1.47) | 0.94 (0.50, 1.77) | 1.67 (0.72, 3.85) | |
| Vigorous | 0.91 (0.50, 1.64) | 0.99 (0.70, 1.40) | 0.91 (0.44, 1.88) | 1.61 (0.64, 4.04) | |
| ^1^ Adjusted odds ratio (aOR) based on univariable proportional odds logistic regression from multivariable proportional odds logistic regression model adjusted for age, sex, study site, obesity, physical activity, cigarette use pack-years, aspirin use, history of polyps, diabetes mellitus, hyperlipidemia, and alcohol use | | | | |  |
